# The relation between incisal guidance angle and the growth and development of temporomandibular joint: a multi-cross-sectional retrospective study

**DOI:** 10.1186/s12903-021-01716-8

**Published:** 2021-07-28

**Authors:** Ying Li, Wenwen Zhou, Yan Wu, Hongwei Dai, Jianping Zhou

**Affiliations:** 1 Stomatological Hospital of Chongqing Medical University, No. 426 Songshi North Road, Chongqing, China; 2 Chongqing Key Laboratory of Oral Diseases and Biomedical Sciences, Chongqing, China; 3 Chongqing Municipal Key Laboratory of Oral Biomedical Engineering of Higher Education, Chongqing, China

**Keywords:** Incisal guidance angle, Temporomandibular joint, Morphology

## Abstract

**Background:**

The incisal guidance angle (IGA) is related to temporomandibular joint (TMJ), and changes to the IGA are often involved in the prosthetic and orthodontic treatment of anterior teeth. However, the influence of incisal guidance on the growth, development and remodelling of the TMJ is not yet clear. The aim of this study was to investigate age-related morphological differences in the TMJ in subjects with different IGAs.

**Methods:**

Cone-beam computed tomography (CBCT) images of 274 patients were included (group 1, IGA < 45°; group 2, 45° ≤ IGA ≤ 60°; group 3, IGA > 60°). Each group was then divided into 4 age groups (group a, 6–12 years; group b, 13–16 years; group c, 17–25 years; group d, 26–33 years). TMJ morphology was assessed by linear measurements, angular measurements, and subjective evaluations. The IGA and occlusal plane angle were also measured.

**Results:**

Anterior inclination of condyle (AIC) increased with age in the three IGA groups but decreased from 17 years onward in group 2 (*P* < 0.05). In the age groups analysis, the AIC in group 1 was smaller than that in group 3 but larger than that in group 2 (*P* > 0.05). Articular eminence inclination (AEI) decreased with age in group 1 (*P* = 0.027) but increased with age in group 3 (*P* = 0.053). The AEI in group 2 was larger than that in group 1 at 17–25 years (*P* = 0.046), and it was larger in group 3 than in group 1 at 26–33 years (*P* = 0.047). The IGA had a weak correlation with AEI (*P* < 0.05).

**Conclusion:**

The articular fossa of patients with shallower incisal guidance changed to a flatter shape with age, whereas the condylar anterior slope and articular eminence of patients with steeper incisal guidance changed towards a steeper alignment. There was a correlation between IGA and TMJ shape.

## Background

It is widely known that the TMJ is one of the most complex joints in the human body. The functional coordination of the TMJ is of great importance in the maintenance of normal masticatory system movement. Several studies have already reported that the complex morphological structure of the TMJ is related to its function [1, 2]. The loads on the mandible and temporomandibular joint differ depending on maxillofacial morphology, and the response of the temporomandibular joint to load change is histological remodelling, such as local tissue hyperplasia or absorption. Anatomical changes can be seen in the parts with obvious reconstruction. Some scholars have examined the differences in the temporomandibular joint related to diverse malocclusions, and confirmed that the morphological structure and positional relationship between the condyle and articular fossa may be different in patients with various types of malocclusion [3, 4].

The incision guidance (IG) is one of the determinants of occlusion [5]. Occlusal factors, including incisal guidance, are often altered during prosthetic and orthodontic treatment, such as occlusal reconstruction and restorative treatment of anterior teeth. Furthermore, children with oligodontia or anodontia often need partial or complete denture restoration at an early age [6, 7], which is likely to impact incisal guidance. Incision and condylar guidance regulate mandibular movements. Shallow anterior guidance guides the mandible to move forward excessively when the mandible is in forward movement, while steep anterior guidance may hinder mandibular advancement. These two malocclusion deformities result in abnormal disc pressure that may affect the anterior slope of the condyle and articular eminence.

Many scholars have reported that IG influences condylar guidance, which in turn modifies TMJ morphology [5, 8]. Different condylar and incisal guidance ratios have been found to affect the activity of anterior and posterior temporal muscles [9]. Schuyler et al. [10] suggested that during growth and development, incisal guidance may influence the contour of the articular fossa and the movement pattern of the condyle. However, no study on this topic has been conducted. Tinastepe et al. [11] found that patients with increased vertical overlap occlusions and minimal horizontal overlap had more clinical symptoms associated with temporomandibular disorders (TMDs) than patients with normal mandibular anatomy. These findings suggest that IG may affect the function of the TMJ, and is an important initiator of TMDs. In adults with limited compensatory ability and slowed TMJ remodelling, abnormal external pressure can easily result in TMJ structural degradation, thus affecting its function.

However, the relevant research data remain still insufficient. To date, several studies have investigated changes in the fossa and condyle in patients of different ages [12, 13]. Katsavrias et al. [14, 15] investigated the growth trajectory of the articular eminence height and inclination. The study of Chae et al. [13] showed no statistically significant differences in the condylar fossa relationship according to age (early, middle, and late adolescence). However, most studies involved few observation points and examined a small age range. Moreover, at present, there are few studies on the influence of the incisal guidance angle on the morphology of the temporomandibular joint. Han et al. [16] revealed weak but statistically significant correlations between the centroid size of the condyle and fossa and the incisal guidance angle (IGA). However, he rejected any correlation between shape variation in the fossa and condyle and the incisal guidance angle. The correlation between these features is still not clear. In general, there has been a lack of research on temporomandibular joint morphology in patients with disparate incisal guidance at different ages.

For on the above reasons, this study attempted to use Dolphin Imaging Version 11.9 (Chatsworth, Calif) to import CBCT images from patients with diverse IGAs and various ages and to use linear measurements, angle measurements and subjective measurements to measure temporomandibular joint morphology and IGAs. The measurement data were analysed to investigate the morphological differences in the TMJ of patients with different incisal guidance angles at different ages to establish a reference for the establishment of incisal guidance. The aim of this study was to evaluate the following null hypothesis: The incisor guidance angle has no effect on the morphologic changes observed in the TMJ with age.

## Methods

### Ethical approval

This study was approved by the Research Ethics Board of the Stomatological Hospital of Chongqing Medical University (No. 2021–003).

### Sample-size calculation

The test level α = 0.05 and test efficiency 1 − β = 0.9 were employed. The predictive value was determined by a pilot experiment, and the sample size required for this study was calculated using PASS (version 15.0, NCSS, LLC). It was determined that 192 participants were required.

### Subjects

Patients (8 to 33 years old) who had initial CBCT (KaVo 3D exam, 120 kV, 5 mA, voxel size of 0.4 mm, field of view of 160 × 170 mm, and 8.9-s scan time) images obtained at the Stomatological Hospital of Chongqing Medical University, Chongqing, China were selected as participants. All images were taken by the same radiographer. The patients were imaged in maximum dental intercuspation in the Frankfort horizontal (FH) plane parallel to the floor. The CBCT images were obtained for several clinical needs (dental implantation, extraction of impacted teeth, third molars resolution, and other clinical examinations).

The study was conducted with 274 patients, 117 males and 157 females, all of whom were normodivergent (FMA 27°–32°). They were classified into 3 groups according to IGA: group 1 (n = 107, IGA < 45°, protrusive upper incisors), group 2 (n = 64, 45° ≤ IGA ≤ 60°), and group 3 (n = 103, IGA > 60°, palatally inclined upper incisors). The participants were then divided into four age groups according to age: group a, 6–12 years (72 patients, 144 joints); group b, 13–16 years (70 patients, 140 joints); group c, 17–25 years (66 patients, 132 joints); and group d, 26–33 years (66 patients, 132 joints).

Exclusion criteria included previous orthodontic treatment, restoration of an anterior tooth, history of dentofacial trauma or temporomandibular disorder, missing incisors or a missing first molar, rheumatoid arthritis or other degenerative joint disease.

### Study design

**Fig. 1:**
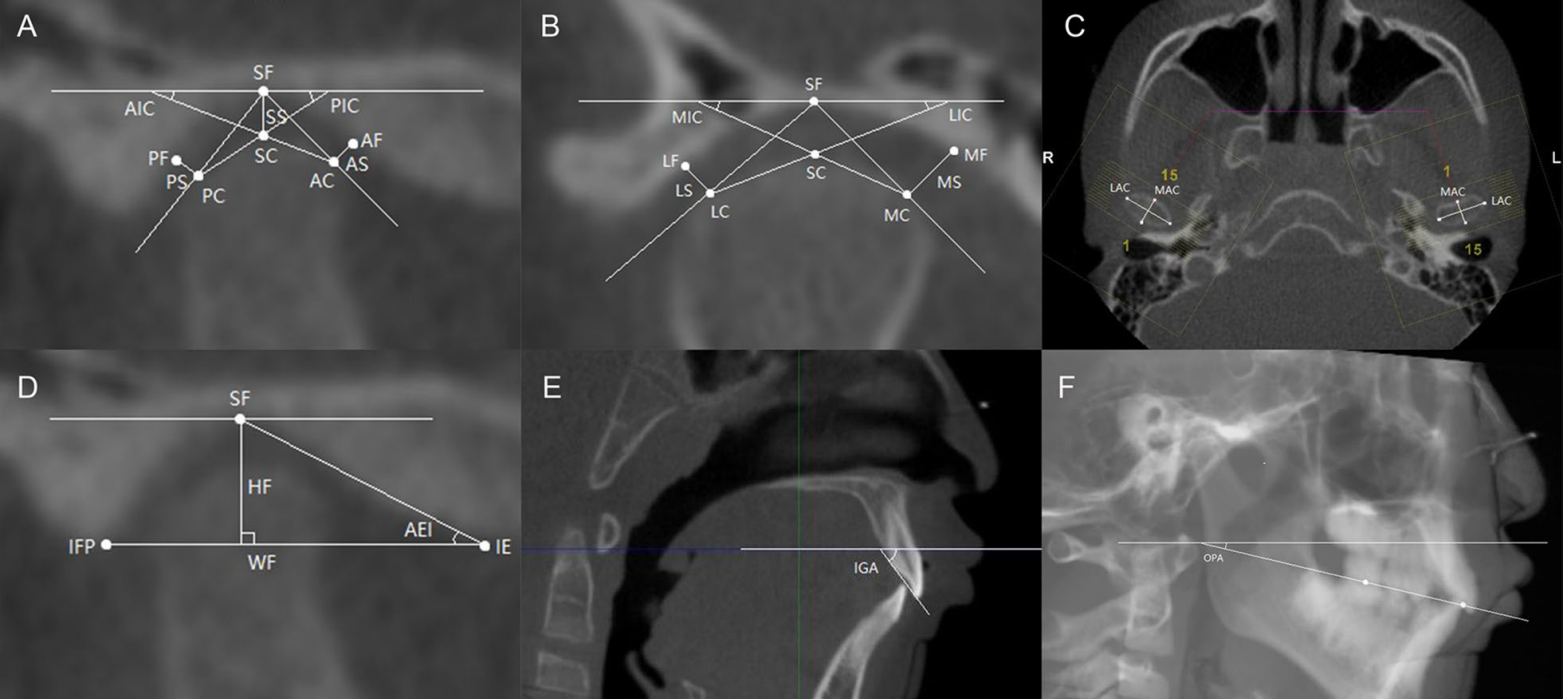
Landmarks and measurements. **A** AF, anterior fossa; AC, anterior condyle; SF, superior fossa; SC, superior condyle; PF, posterior fossa; PC, posterior condyle; SS, superior space; AS, anterior space; PS, posterior space; AIC, anterior inclination of the condyle; PIC, posterior inclination of the condyle. **B** MF, medial fossa; MC, medial condyle; LF, lateral fossa; LC, lateral condyle; MS, medial space; LS, lateral space; MIC, medial inclination of the condyle; LIC, lateral inclination of the condyle. **C** LAC, long axis of the condyle; MAC, minor axis of the condyle. **D** IE, inferior eminence; IFP, inferior fossa posterior wall; HF, height of the fossa; WF, width of the fossa; AEI, articular eminence inclination. **E** IGA, incisal guidance angle. **F** OPA, occlusal plane angle

The Frankfort horizontal (FH) plane, which was constructed by Orbitale on the right side and by Porion on both sides, was used as the horizontal reference plane for the reconstructed images [13]. The left and right joints were evaluated separately. Dolphin Imaging Version 11.9 (Chatsworth, CA) was used to digitize landmarks. Morphological evaluation of the TMJ was performed using linear measurements, angular measurements, and subjective evaluation. The slices that showed the greatest anteroposterior and mediolateral dimensions of the condylar head were selected on the sagittal, coronal and transverse views, respectively. The basic condylar shapes used for classification were round, oval, flat, and triangular; the basic fossa shapes were round, oval, triangular, and trapezoidal. The landmarks and linear and angular measurements used for the analysis are as follows:

Condyle (Fig. 1A–C): The TMJ space is the shortest distance between 2 points or 2 lines: anterior space (AS), anterior fossa (AF), and anterior condyle (AC); superior space (SS), the horizontal lines through the superior fossa (SF) and superior condyle (SC); posterior space (PS), posterior fossa (PF), and posterior condyle (PC); medial space (MS), medial fossa (MF), and medial condyle (MC); lateral space (LS), lateral fossa (LF), and lateral condyle (LC). The anterior inclination of the condyle (AIC) is the angle between the line connecting the superior condyle (SC) and anterior condyle (AC) and the FH plane. The posterior inclination of the condyle (PIC) is the angle between the line connecting the superior condyle (SC) and posterior condyle (PC) and the FH plane. The medial inclination of the condyle (MIC): the angle between the line connected superior condyle (SC) and medial condyle (MC) and FH plane. The lateral inclination of the condyle (LIC) is the angle between the line connecting the superior condyle (SC) and lateral condyle (LC) and the FH plane. The long axis of the condyle (LAC) is the greatest mediolateral diameter of the condyle. The minor axis of the condyle (MAC) is the greatest anteroposterior diameter of the condyle.

Fossa (Fig. 1D): The articular eminence inclination (AEI) is The angle between the line connecting the superior fossa (SF) and inferior eminence (IE) and the line connecting the inferior eminence (IE) and inferior fossa posterior wall (IFP). The height of the fossa (HF) is the vertical distance from the superior fossa (SF) to the line connecting the inferior eminence and the inferior fossa posterior wall (IFP). The width of the fossa (WF) is the shortest distance between the inferior eminence (IE) and inferior fossa posterior wall (IFP).

The incisal guidance angle (IGA) (Fig. 1E) is the angle between the line connecting the incisal margin of the maxillary and mandibular incisors and the FH plane. The higher value of the two central incisors was selected [9, 16].

To measure the occlusal plane angle (OPA) (Fig. 1F), the line connecting the midpoint of the overbite of the central incision and the midpoint of the overbite of the first molar was taken as the occlusal plane. The angle formed between the occlusal plane and the FH plane was then measured [17].

The IGA and OPA were measured in the midsagittal plane. The AS, SS, PS, AEI, HF, WF, AIC, PIC, MIC, and LIC were measured on the median sagittal slice which was perpendicular to the long axis of the condyle. The MS, LS, MIC, and LIC were measured on the median coronary slice which was parallel to the long axis of the condyle. The LAC and MAC were measured on the transverse plane of the condyle.

All parameters were measured three times by two researchers at a 2-week interval. The average values of the six measurements were taken for statistical analysis.

### Statistical analysis

Statistical evaluation was performed using IBM SPSS Statistics, version 26.0 (IBM Corp, USA). Normal distribution was confirmed using the ShapiroWilk test. The right and left variables were compared using a paired t-test, and then averaged for further analysis because no significant differences were observed. Percentages of observed shapes were calculated. Two-way multivariate analysis of variance (two-way MANOVA) was performed to compare the intergroup differences. A linear trend test (P for trend) was performed to analyse the trend of each IGA group with age. Pearson correlation analysis was performed to evaluate the correlation between the incisal guidance angle (IGA) and occlusal plane angle (OPA) and their respective correlations with measurement items related to the morphology of the articular fossa and condyle. The significance level was set at 0.05.

## Result

### Correlation analysis of the IGA and OPA

There was a positive correlation between the OPA and IGA (*P* = 0.000, r = 0.381), which also had a weak correlation with articular eminence inclination (AEI) (*P* = 0.016, r = 0.145) and lateral inclination of the condyle (LIC) (*P* = 0.022, r = − 0.139). The OPA and anterior inclination of the condyle (AIC) were negatively correlated, but the correlation was weak (*P* = 0.001, r = − 0.201) (Table 1).

**Table 1:** Correlation analysis on IGA and OPA

	IGA		OPA	
	r	*P*	r	*P*
Incisal guidance angle (IGA)	–	–	0.381	0.000**
Occlusal plane angle (OPA)	0.381	0.000**	–	–
Medial space (MS)	− 0.182	0.003**	− 0.338	0.000**
Lateral space (LS)	− 0.114	0.059	− 0.149	0.014*
Medial inclination of the condyle (MIC)	− 0.085	0.160	− 0.072	0.237
Lateral inclination of the condyle (LIC)	− 0.139	0.022*	0.036	0.548
Superior space (SS)	− 0.124	0.041*	− 0.342	0.000**
Anterior space (AS)	− 0.005	0.930	− 0.096	0.113
Posterior space (PS)	− 0.141	0.019*	− 0.185	0.002**
Anterior inclination of the condyle (AIC)	0.054	0.370	− 0.201	0.001**
Posterior inclination of the condyle(PIC)	0.008	0.898	− 0.002	0.976
Height of the fossa (HF)	0.108	0.073	− 0.036	0.548
Width of the fossa (WF)	0.071	0.241	− 0.029	0.628
Articular eminence inclination (AEI)	0.145	0.016*	− 0.013	0.827
Long axis of the condyle (LAC)	0.057	0.350	− 0.410	0.000**
Minor axis of the condyle (MAC)	− 0.027	0.653	− 0.085	0.161

*P < 0.05

**P < 0.01

### Condylar morphology

The anterior space (AS) tended to increase with age in group 3 (*P* = 0.003) (Tables 2, 3). The largest AS in the three IGA groups was found in group 3 after the age of 6–12 years (Tables 2, 4).

**Table 2:** Descriptive statistics for variables in each age group

	1				2				3			
	6–12	13–16	17–25	26–33	6–12	13–16	17–25	26–33	6–12	13–16	17–25	26–33
Incisal guidance angle(IGA)	37.47 ± 6.73	34.47 ± 6.34	35.81 ± 7.00	37.34 ± 6.09	50.62 ± 3.90	49.66 ± 3.14	51.06 ± 4.62	48.16 ± 3.17	69.11 ± 6.95	68.15 ± 5.37	69.31 ± 7.84	72.07 ± 7.10
Occlusal plane angle (OPA)	6.96 ± 4.50	3.70 ± 5.27	2.93 ± 4.47	2.88 ± 3.56	12.68 ± 4.56	8.10 ± 3.01	7.81 ± 3.21	5.60 ± 2.73	11.01 ± 4.02	8.06 ± 4.84	4.63 ± 4.58	5.78 ± 4.02
Medial space (MS)	2.47 ± 0.67	2.30 ± 0.60	2.70 ± 1.20	2.56 ± 0.62	1.83 ± 0.60	1.95 ± 0.60	2.22 ± 0.69	2.46 ± 0.71	2.09 ± 0.53	2.21 ± 0.68	2.32 ± 0.61	2.29 ± 0.62
Lateral space (LS)	2.17 ± 0.43	1.95 ± 0.40	1.92 ± 0.60	2.16 ± 0.61	1.87 ± 0.59	1.56 ± 0.44	2.04 ± 0.66	1.88 ± 0.50	1.91 ± 0.50	1.92 ± 0.44	1.96 ± 0.43	1.93 ± 0.52
Medial inclination of the condyle (MIC)	14.83 ± 3.29	14.96 ± 3.42	16.48 ± 6.35	17.05 ± 3.77	15.97 ± 4.86	17.67 ± 2.92	16.10 ± 3.58	15.30 ± 4.01	14.26 ± 2.67	15.59 ± 4.07	15.12 ± 4.61	14.79 ± 4.14
Lateral inclination of the condyle (LIC)	18.94 ± 4.86	18.12 ± 4.29	18.62 ± 4.89	19.01 ± 4.42	16.80 ± 4.28	18.13 ± 3.05	17.78 ± 4.83	18.96 ± 5.15	16.09 ± 5.20	18.32 ± 3.89	14.84 ± 4.11	17.72 ± 5.05
Superior space (SS)	2.83 ± 0.65	2.80 ± 0.46	2.87 ± 0.80	3.16 ± 0.63	2.59 ± 0.65	2.38 ± 0.60	2.95 ± 0.75	2.94 ± 0.59	2.48 ± 0.40	2.76 ± 0.42	2.91 ± 0.62	2.97 ± 0.65
Anterior space (AS)	1.84 ± 0.43	1.65 ± 0.41	1.88 ± 0.56	1.95 ± 0.55	1.51 ± 0.48	1.61 ± 0.47	1.81 ± 0.39	1.81 ± 0.55	1.47 ± 0.39	1.83 ± 0.65	1.95 ± 0.79	2.02 ± 0.76
Posterior space (PS)	2.05 ± 0.54	1.70 ± 0.44	1.74 ± 0.52	1.81 ± 0.47	1.73 ± 0.60	1.36 ± 0.26	1.64 ± 0.42	1.64 ± 0.34	1.69 ± 0.40	1.73 ± 0.38	1.66 ± 0.41	1.73 ± 0.44
Anterior inclination of the condyle (AIC)	25.17 ± 6.71	28.41 ± 6.51	29.39 ± 6.15	31.12 ± 5.09	23.58 ± 5.07	28.04 ± 5.34	29.26 ± 4.08	29.13 ± 4.04	25.78 ± 4.80	28.62 ± 6.17	30.81 ± 6.41	32.68 ± 5.50
Posterior inclination of the condyle(PIC)	25.64 ± 7.02	24.00 ± 4.57	25.59 ± 5.35	25.91 ± 4.94	25.17 ± 5.50	26.47 ± 3.11	25.52 ± 4.15	28.10 ± 4.43	25.18 ± 4.78	25.85 ± 4.59	25.14 ± 4.15	25.86 ± 4.87
Height of the fossa (HF)	6.40 ± 0.91	6.64 ± 1.36	6.10 ± 1.12	6.17 ± 0.80	5.83 ± 0.84	6.60 ± 0.67	6.96 ± 0.96	6.33 ± 0.82	6.21 ± 1.02	6.74 ± 0.97	6.63 ± 1.00	6.54 ± 0.77
Width of the fossa (WF)	16.57 ± 1.46	16.76 ± 1.85	16.88 ± 1.97	16.69 ± 1.15	16.51 ± 1.40	17.19 ± 1.46	16.94 ± 1.49	16.25 ± 1.46	16.49 ± 1.98	17.46 ± 1.51	17.04 ± 1.49	16.72 ± 1.64
Articular eminence inclination (AEI)	35.01 ± 5.58	32.57 ± 5.16	31.64 ± 6.70	31.70 ± 4.68	31.35 ± 4.09	33.85 ± 4.55	35.94 ± 7.14	33.14 ± 4.89	32.74 ± 5.16	33.46 ± 5.48	34.74 ± 6.70	35.48 ± 4.78
Long axis of the condyle (LAC)	16.43 ± 1.46	17.97 ± 2.17	18.33 ± 2.30	18.88 ± 1.63	16.34 ± 2.46	18.56 ± 1.81	19.78 ± 1.94	19.32 ± 1.93	16.91 ± 1.92	18.18 ± 1.70	19.56 ± 2.78	19.23 ± 1.91
Minor axis of the condyle (MAC)	8.35 ± 0.77	8.83 ± 0.87	9.15 ± 0.76	9.32 ± 0.84	9.45 ± 1.12	9.02 ± 0.63	9.45 ± 0.63	9.18 ± 0.76	8.64 ± 0.81	8.83 ± 0.87	8.96 ± 0.79	9.06 ± 0.87

1: IGA < 45°; 2: 45° ≤ IGA ≤ 60°; 3: IGA > 60°

**Table 3:** Linear term in trend test among three IGA groups

Variable	1		2		3	
	F	P	F	P	F	P
Occlusal plane angle (OPA)	10.936	0.001**	31.097	0.000**	25.069	0.000**
Medial space (MS)	0.934	0.336	8.602	0.005**	1.819	0.181
Lateral space (LS)	0.017	0.897	0.662	0.419	0.083	0.774
Medial inclination of the condyle (MIC)	4.703	0.032*	0.670	0.416	0.106	0.745
Lateral inclination of the condyle (LIC)	0.032	0.859	1.543	0.219	0.127	0.722
Superior space (SS)	3.505	0.064	4.837	0.032*	12.102	0.001**
Anterior space (AS)	1.705	0.195	4.248	0.044*	9.361	0.003**
Posterior space (PS)	2.510	0.116	0.000	0.984	0.020	0.887
Anterior inclination of the condyle (AIC)	12.293	0.001**	11.705	0.001**	20.947	0.000**
Posterior inclination of the condyle (PIC)	0.251	0.617	2.546	0.116	0.108	0.743
Height of the fossa (HF)	1.678	0.198	4.024	0.049*	1.175	0.281
Width of the fossa (WF)	0.104	0.748	0.402	0.529	0.033	0.857
Articular eminence inclination (AEI)	5.020	0.027*	1.585	0.213	3.827	0.053
Long axis of the condyle (LAC)	21.250	0.000**	19.561	0.000**	20.465	0.000**
Minor axis of the condyle (MAC)	20.897	0.000**	0.172	0.679	3.594	0.061

1: IGA < 45°; 2: 45° ≤ IGA ≤ 60°; 3: IGA > 60°

*P < 0.05

**P < 0.01

**Table 4:** Statistical significance was found in pairwise comparisons

	1			2			3			6–12		13–16		17–25	26–33
OPA	a v b	a v c	a v d	a v b	a v c	a v d	a v c	a v d	b v c	1 v 2	1 v 3	1 v 2	1 v 3	1 v 2	1 v 3
MS										1 v 2					
LS															
MIC															
LIC							b v c							1 v 3	
SS							a v d								
AS							a v c	a v d		1 v 3					
PS	a v b									1 v 3		2 v 3			
AIC	a v c	a v d		a v c	a v d		a v c	a v d							
PIC															
HF				a v c										1 v 2	
WF															
AEI														1 v 2	1 v 3
LAC	a v b	a v c	a v d	a v b	a v c	a v d	a v c	a v d							
MAC	a v c	a v d								1 v 2	2 v 3				

1: IGA < 45°; 2: 45° ≤ IGA ≤ 60°; 3: IGA > 60°

a: 6–12 years old group; b: 13–16 years old group; c: 17–25 years old group; d: 26–33 years old group

*OPA* occlusal plane angle, *MS* medial space, *LS* lateral space, *MIC* medial inclination of the condyle, *LIC* lateral inclination of the condyle, *SS* superior space, *AS* anterior space, *PS* posterior space, *AIC* anterior inclination of the condyle, *PIC* posterior inclination of the condyle, *HF* height of the fossa, *WF* width of the fossa, *AEI* articular eminence inclination, *LAC* long axis of the condyle, *MAC* minor axis of the condyle

**Fig. 2:**
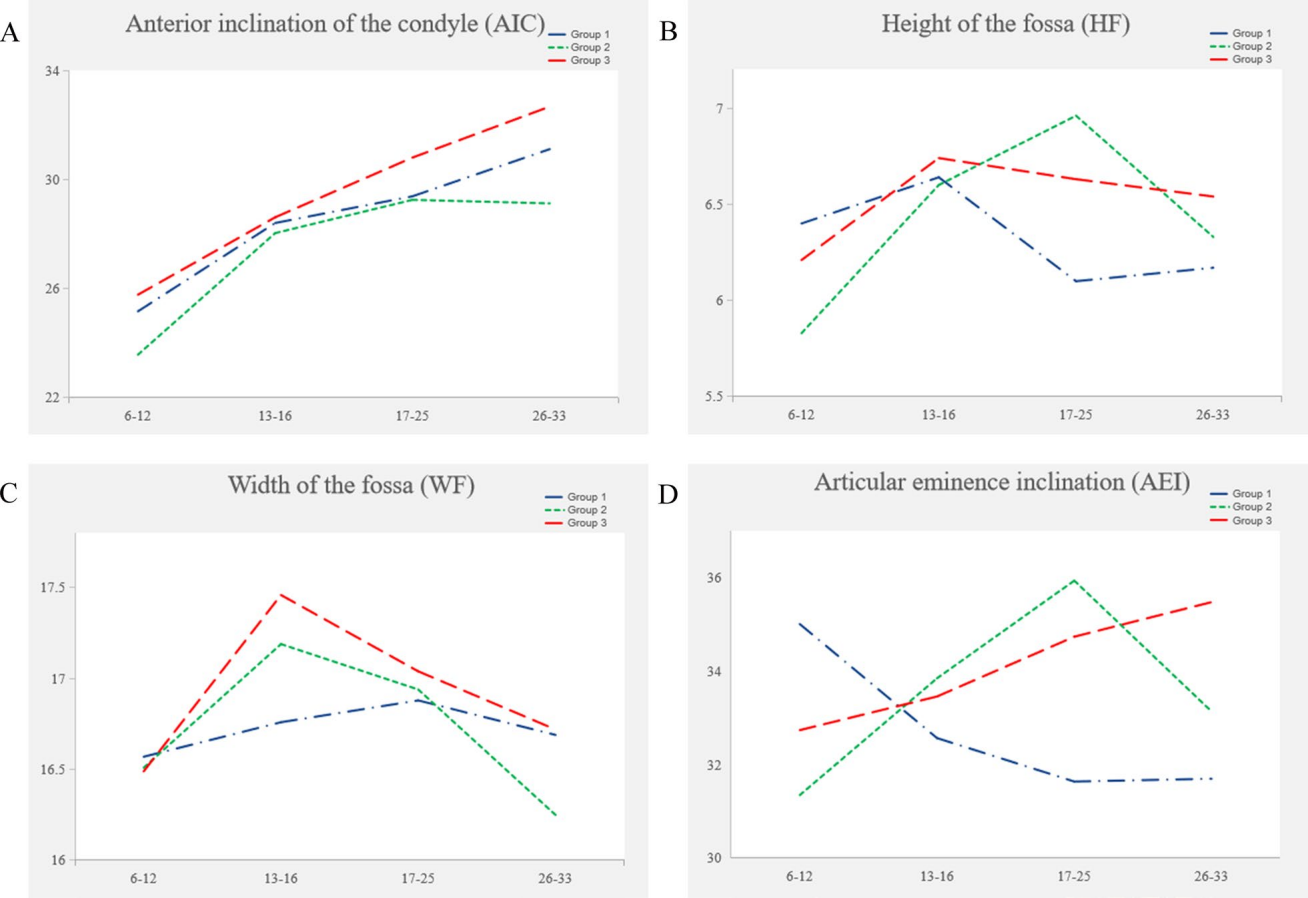
Trends of different IGA groups with age. **A** Anterior inclination of the condyle (AIC); **B** Height of the fossa (HF); **C** Width of the fossa (WF); **D** Articular eminence inclination (AEI)

Anterior inclination of the condyle (AIC) tended to increase with age in the three IGA groups, but to slightly decrease from 17 years old onward in group 2 (*P* < 0.05). In the age group analysis, the AIC in group 1 was smaller than that in group 3 but larger than that in group 2 (*P* > 0.05) (Fig. 2A, Tables 2, 3).

The long axis of the condyle (LAC) tended to increase with age in group 1 (*P* = 0.000), and to increase with age from 6 years old and then decrease with age after 17 years of age in groups 2 and 3 (*P* < 0.05). From 13 to 16 years old, the LAC in group 2 was larger than in group 3, while the LAC in group 3 was larger than that in group 1 (*P* > 0.05) (Tables 2, 3, 4).

The minor axis of the condyle (MAC) tended to increase with age in group 1 (*P* = 0.000). At 6–12 years old, the MAC in group 2 was larger than that in groups 1 and 3 (*P* < 0.05) (Tables 2, 3, 4).

### Fossa morphology

The height of the fossa (HF) tended to increase from 6–12 years to 17–25 years, and then decrease with age in group 2 (*P* = 0.049) (Fig. 2B, Tables 2, 3). The only significant HF difference between the 6–12-year-old group and the 17–25-year-old group existed in group 2 (*P* = 0.007). The HF in group 1 was significantly smaller than that in group 2 in the 17–25-year-old group (*P* = 0.021) (Tables 2, 4).

The average width of the fossa (WF) increased first and then decreased with age (*P* > 0.05) (Fig. 2C, Tables 2, 3). The turning point of group 1 (17–25 years) was different from that of the other two IGA groups (13–16 years). However, there was no significant difference in the pairwise comparison (Table 4).

Articular eminence inclination (AEI) tended to decrease with age in group 1 (*P* = 0.027). It increased with age in group 3 and group 2 but decreased from 17 years of age onward in group 2 (*P* > 0.05) (Fig. 2D, Tables 2, 3). The AEI in group 2 was larger than that in group 1 in the 17–25 year-old group (*P* = 0.046). It was larger in group 3 than in group 1 in the 26–33 year-old group (*P* = 0.047) (Tables 2, 4).

### OPA

Pairwise comparisons suggested that the occlusal plane angle (OPA) changed significantly at different age groups (*P* < 0.05) and it tended to decrease with age (*P* < 0.05) (Tables 2, 3, 4).

### Subjective evaluation of fossa and condylar shape

In the three IGA groups, the oval shape was the most common shape of the condyle and fossa. In the 6–12-year age group, the shape of the articular fossa was mostly round, although it was mostly oval in the other age groups. The proportion of oval-shaped condyles and fossae increased with age (Table 5).

**Table 5:** Percentage of condylar and fossa observed shapes

	1		2		3		6–12		13–16		17–25		26–33	
	Condyle	Fossa	Condyle	Fossa	Condyle	Fossa	Condyle	Fossa	Condyle	Fossa	Condyle	Fossa	Condyle	Fossa
Round	27.1	26.6	22.7	37.5	14.6	27.2	44.4	52.1	20	30	9.1	19	9.8	14.4
Oval	66.8	61.7	76.5	55.5	81.5	55.3	52.8	36.8	77.9	60	85.6	63.7	84.1	72.7
Trapezoidal	–	7	–	3.1	–	11.7	–	10.4	–	6.4	–	9.8	–	4.5
Triangular	4.7	4.7	0.8	3.9	2.9	5.8	0.7	0.7	1.4	3.6	4.5	7.5	6.1	8.4
Flattened	1.4	–	–	–	1	–	2.1	–	0.7		0.8	–	–	–

1: IGA < 45°; 2: 45° ≤ IGA ≤ 60°; 3: IGA > 60°

## Discussion

This study comprised on multiple simultaneous cross-sectional studies on the morphology of the TMJ with different incisal guidance. In this study, subjects were divided into four age groups to explore the changes in the morphology of the TMJ with age. Our findings suggest that morphology of TMJs under different incisal guidance is dissimilar, and changes differently with age. Therefore, the null hypothesis of this study was rejected.

Many scholars have supported the premise that the shape of the temporomandibular joint is related to its function, and the temporomandibular joint load varies with dentofacial morphology [2, 3, 18, 19]. Pullinger et al. [20] reported that in comparison to Class I patients, Class II patients had more condylar positions that were not in the middle, and Class II Division 1 malocclusion patients often had anterior displacement of the condyle. Katsavrias et al. [21] measured the shape of the condylar fossa in Class II Division 2 patients and found that the condylar position changed from anterior to posterior with age. These data are consistent with our findings.

Regarding the change in the occlusal surface with age, a growth study conducted in children aged 3 months to 8 years revealed that the angle of the occlusal surface to the palatal plane, SN plane, and mandibular plane remained unchanged [22, 23]. However, in a study of children aged 8–17 years, the occlusal surface was found to be less inclined and more parallel with growth [24]. These conclusions support our results, which showed the same trend: after the age of 8, the OPA gradually decreases with age.

With respect to the anterior inclination of the condyle (AIC), we found that the AIC increases with age. The AIC of group 3 patients was the largest, although there was no significant difference between the IGA groups. This may be due to remodelling of the condyle with age. A larger incisal guidance angle may make the anterior slope of the condyle steeper. We also observed that the long axis of the condyle (LAC) in group 2 is larger than that in groups 1 and 3 in all age groups. We can speculate that abnormal incisal guidance may hinder the growth and development of the condyle. A possible explanation is that abnormal incisal guidance may cause abnormal pressure in the TMJ disc, which has been proven to influence the proliferation and matrix synthesis of condylar chondrocytes [25].

Katsavrias et al. [15] reported that the articular eminence height grows rapidly before the age of 7 (the deciduous dentition), that growth is almost non-existent between 7 and 11 years (the mixed dentition), and that the remaining height is achieved by age 20. Sülün et al. [12] reported that the eminence inclination reaches a maximum value between 21 and 30 years old in healthy patients and decreases after 31 years of age. In our study, we also observed that the height of the fossa (HF) increased after 6 years of age and then decreased after age 17 in group 2. Early increases in the fossa height are theorized to be caused by growth and development, and the later decrease may be related to the remodelling of the articular fossa. The finding that the HF in group 1 was lowest after the peak of growth and development suggests that patients with shallower incisal guidance have a flatter fossae.

It has been suggested that steep incisal guidance could result in abnormal movements in the TMJ [26]. Dawson et al. [27] contends that the shape of the TMJ is determined by the interaction of anterior guidance, the occlusal surface and TMJ morphology, which could be affected by occlusion. Anterior guidance could be linked not only to early condylar movement but also to the size and path of the condyle and fossa. Han et al. [16] found a correlation between the centroid size of the condyle and fossa and the incisal guidance angle. The incisal guidance angle and the occlusal plane angle were also correlated. Our results support this conclusion. However, they maintained that the shape change of the fossa and condyle had nothing to do with the occlusal plane angle and the incisal guidance angle. In our study, the results of the correlation analysis showed that there was a weak correlation between the incisal guidance angle (IGA) and fossa morphology. With regard to articular eminence inclination (AEI), we found that it had a tendency to decrease with age in group 1. The other two IGA groups had different trends, but they were not statistically significant. In group 2, AEI increased from the age of 6 and then decreased after the age of 17. However, in group 3, AEI increased with age. A larger sample size may be needed to confirm this. The reason for the different trends may be due to different incisal guidance angles affecting the motion trajectory of the mandible differently. A small incisal guidance angle might guide the mandible to move forward excessively during forward movement., while a large incisal guidance angle might hinder the protrusion of the mandibular, which will cause changes in the contractile force of the masticatory muscles through the neuromuscular reflex, thus changing the load of the TMJ, which is an important reason for changes in the remodelling of the TMJ. In addition, after the peak period of growth and development, the AEI in group 1 was smaller than that in IGA groups 2 and 3, which was consistent with a previous finding that the HF was the lowest in group 1. This finding suggests a flatter fossa shape, which may be related to the remodelling of the articular fossa, and the reason for the remodelling towards a flatter shape may be the smaller incisal path inclination. In short, we can speculate that the incisal guidance angle has a certain guiding effect on the shape of TMJ reconstruction.

These results suggest that if a patient's previous incisal guidance is not known, the clinician can select the incisal guidance angle according to the shape of the temporomandibular joint. Shallow incisal guidance might be suitable for patients with flat fossa, and a large incisal guidance angle might be suitable for patients with steep and deep fossa. The incisors should also be considered in the diagnosis and treatment of TMDs.

Several limitations of the study are worth noting. We analysed morphological changes of the TMJ in four age groups and found that the TMJs of most patients began to show significant morphological changes from the age of 17, but this needs to be further confirmed with analyses of more age groups in future studies. However, this did not prevent us from reaching a new conclusion. In addition, all of our studies focused on patients with incisal guidance, and we could also study patients without incisal guidance, such as those with an anterior crossbite or open bite.

## Conclusion

Patients with different incisal guidance have different condylar and articular fossa shapes, and different trends with age. Patients with more shallow incisal guidance tended to have a flatter fossa with age, whereas patients with steeper incisal guidance had a tendency towards a steeper anterior condylar slope and increased articular eminence inclination.

After the peak period of growth and development, the TMJ gradually shows different morphological characteristics related to the IGA after the age of 17.

The incisal guidance angle had a weak correlation with TMJ morphology.

## Data Availability

The data underlying this article will be shared on reasonable request to the corresponding author.

## Abbreviations

IGA: incisal guidance angle
TMJ: temporomandibular joint
CBCT: cone-beam computed tomography
IG: incision guidance
TMDs: temporomandibular disorders
FH plane: frankfort horizontal plane
AF: anterior fossa
AC: anterior condyle
SF: superior fossa
SC: superior condyle
PF: posterior fossa
PC: posterior condyle
SS: superior space
AS: anterior space
PS: posterior space
AIC: anterior inclination of the condyle
PIC: posterior inclination of the condyle
MF: medial fossa
MC: medial condyle
LF: lateral fossa
LC: lateral condyle
MS: medial space
LS: lateral space
MIC: medial inclination of the condyle
LIC: lateral inclination of the condyle
LAC: long axis of the condyle
MAC: minor axis of the condyle
IE: inferior eminence
IFP: inferior fossa posterior wall
HF: height of the fossa
WF: width of the fossa
AEI: articular eminence inclination.
IGA: incisal guidance angle.
OPA: occlusal plane angle.
Two-way MANOVA: two-way multivariate analysis of variance
